# Gender differences in patient journey to diagnosis and disease outcomes: results from the European Map of Axial Spondyloarthritis (EMAS)

**DOI:** 10.1007/s10067-020-05558-7

**Published:** 2021-01-19

**Authors:** Marco Garrido-Cumbrera, Denis Poddubnyy, Laure Gossec, Raj Mahapatra, Christine Bundy, Souzi Makri, Sergio Sanz-Gómez, Laura Christen, Carlos J. Delgado-Domínguez, Victoria Navarro-Compán, on behalf of the EMAS Working Group

**Affiliations:** 1 Health & Territory Research (HTR), Universidad de Sevilla, Seville, Spain; 2 Axial Spondyloarthritis International Federation (ASIF), London, Spain; 3 Charité-Universitätsmedizin Berlin, Berlin, Germany; 4 German Rheumatism Research Centre, Berlin, Germany; 5 Institut Pierre Louis d’Epidémiologie et de Santé Publique (iPLESP), Sorbonne Université, Paris, France; 6 AP-HP, Rheumatology Department, Pitié Salpêtrière Hospital, Paris, France; 7 Axial Spondyloarthritis International Federation (ASIF), London, UK; 8 Cardiff University, Cardiff, UK; 9 Cyprus League Against Rheumatism, Nicosia, Cyprus; 10 Novartis Pharma AG, Basel, Switzerland; 11 IdiPaz, University Hospital La Paz, Madrid, Spain

**Keywords:** Axial spondyloarthritis, Europe, Gender, Patient journey, Patient-reported outcomes

## Abstract

**Introduction/objectives:**

To evaluate the journey to diagnosis, disease characteristics and burden of disease in male and female patients with axial spondyloarthritis (axSpA) across Europe.

**Method:**

Data from 2846 unselected patients participating in the European Map of Axial Spondyloarthritis (EMAS) study through an online survey (2017–2018) across 13 countries were analysed. Sociodemographic characteristics, lifestyle, diagnosis, disease characteristics and patient-reported outcomes (PROs) [disease activity –BASDAI (0–10), spinal stiffness (3–12), functional limitations (0–54) and psychological distress (GHQ-12)] were compared between males and females using chi-square (for categorical variables) and student *t* (for continuous variables) tests.

**Results:**

In total, 1100 (38.7%) males and 1746 (61.3%) females participated in the EMAS. Compared with males, females reported considerable longer diagnostic delay (6.1 ± 7.4 vs 8.2 ± 8.9 years; *p* < 0.001), higher number of visits to physiotherapists (34.5% vs 49.5%; *p* < 0.001) and to osteopaths (13.3% vs 24.4%; *p* < 0.001) before being diagnosed and lower frequency of HLA-B27 carriership (80.2% vs 66.7%; *p* < 0.001). In addition, females reported higher degree of disease activity in all BASDAI aspects and greater psychological distress through GHQ-12 (4.4 ± 4.2 vs 5.3 ± 4.1; *p* < 0.001), as well as a greater use of alternative therapies.

**Conclusion:**

The patient journey to diagnosis of axSpA is much longer and arduous in females, which may be related to physician bias and lower frequency of HLA-B27 carriership. Regarding PROs, females experience higher disease activity and poorer psychological health compared with males. These results reflect specific unmet needs in females with axSpA needing particular attention.

**Table:** 

**Key Points** *• Healthcare professionals’ perception of axSpA as a predominantly male disease may introduce some bias during the diagnosis and management of the disease. However, evidence about male-female differences in axSpA is scarce.* *• EMAS results highlight how female axSpA patients report longer diagnostic delay and higher burden of the disease in a large sample of 2846 participants of 13 European countries.* *• Results reflect unmet needs of European female patients. Healthcare professionals should pay close attention in order to accurately diagnose and efficiently manage axSpA cases while further research should be developed on the cause of reported gender differences.*

**Supplementary Information:**

The online version contains supplementary material available at 10.1007/s10067-020-05558-7.

## Introduction

Axial spondyloarthritis (axSpA) is an inflammatory chronic disease of insidious course characterised by recurrent episodes of pain and inflammation that mostly affect the spine and pelvis. Currently, axSpA comprises patients with non-radiographic axSpA (nr-axSpA) and radiographic axSpA (r-axSpA, also known as ankylosing spondylitis, AS) [1]. There is a growing body of research revealing that the disease manifests differently in female and male patients due to gender variations of the immunological, hormonal and genetic responses [2]. However, many of the acting mechanisms and consequences remain unclear.

A recent meta-analysis showed that the diagnostic delay in axSpA is longer for females compared with males (8.8 vs 6.5 years) [3]. The reasons for this difference are not completely understood. However, results from a Spanish study carried out at Alicante University General Hospital on 150 people with spondyloarthritis showed that despite men and women reporting the same symptoms for chronic back pain; in two thirds of the healthcare centres, only men were referred to a rheumatology unit [4]. The results from this research seem to suggest a possible bias among physicians, who may think of axSpA only in male patients. This is understandable as historically, the estimated prevalence of axSpA was much higher in men than in women. Initial studies showed a male-female relationship of 10:1 [5, 6], but as research progressed, this gender difference was reduced to 3:1 [7, 8] and even 2:1 [9]. This has been nuanced by research concluding that even that if this sex ratio of male prevalence is generally 2–3:1, numbers could balance when examining only nr-axSpA [10]. However, healthcare professionals’ remaining perception of axSpA as a predominantly male disease may introduce some bias during the diagnosis and follow-up of the disease [3].

Another possible reason explaining why females are diagnosed later could be relevant differences in clinical manifestations or complementary test findings between genders. For example, women tend to have more arthritis than enthesitis-related symptomatology [11, 12], and male patients are more prone to test positive in both HLA-B27 genetic blood testing and imaging techniques (X-SIJ and/or MRI-SIJ) [13].

Furthermore, several publications have shown that there are gender differences in the disease course. Women systematically report a higher burden of disease, including higher BASDAI, total back pain score, [14] and more articular pain, both axial and peripheral [15]. Females also score lower in quality of life measures, have lower response rate to anti-TNF and show lower drug adherence [2]. Additionally, it has also been suggested that female patients undergo more clinical adverse events than males during the course of the disease [16].

Although these previous studies provide valuable insights, most have assessed gender differences focusing on specific aspects of the disease experience, and primarily through a clinical lens. The European Map of Axial Spondyloarthritis (EMAS) study aimed to generate evidence on patient-reported aspects of axSpA using a questionnaire developed in collaboration with patients, the Axial Spondyloarthritis International Federation (ASIF) and clinical academic experts, describing how patients self-reporting as axSpA experience their disease from a physical, psychological and social perspective and how they are managed within healthcare systems.

Using the data from EMAS, the objective of this analysis is to compare gender differences within the journey to diagnosis, disease characteristics and burden of disease including psychological distress.

## Materials and method

### The EMAS working group

The EMAS project is an international initiative promoted by the Axial Spondyloarthritis International Federation (ASIF) and the Spanish Federation of Spondyloarthritis Patient Associations (CEADE), led by the Health & Territory Research group of the University of Seville (HTR) and a steering committee composed of patient representatives and internationally recognised rheumatologists, psychologists and researchers specialised in axSpA.

### Design and survey development

EMAS was an observational, cross-sectional survey of unselected patients self-reporting as axSpA from Austria, Belgium, France, Germany, Italy, the Netherlands, Norway, Russia, Slovenia, Spain, Sweden, Switzerland and the UK. The survey questionnaire was adapted from the Spanish Atlas of Axial Spondyloarthritis 2017 [17], a patient survey held from January to March 2016 promoted by Health and Territory Research and CEADE, with the support of the Max Weber Institute and Novartis Farmacéutica Spain. The data from the Atlas of Axial Spondyloarthritis in Spain 2017 [18] was retrospectively added to the EMAS database.

The final patient questionnaire included 108 items related to 12 different areas: sociodemographic and anthropometric characteristics, disability and performance, work life, daily life, lifestyle habits, diagnostic process, healthcare resource use, treatment, concomitant diseases, extra-articular manifestations, psychological health, disease outcomes and patient experience of living with the disease. All information collected by the EMAS survey was patient-reported and not validated through medical records.

### Supplementary indices

The following supplementary measures were collected in the questionnaire to assess specific areas:

- BASDAI (Bath Ankylosing Spondylitis Disease Activity Index): a validated self-administered questionnaire assessing disease activity in patients with axSpA. Possible scores range from 0 (no activity) to 10 (maximum activity) [19]
- GHQ-12 (General Health Questionnaire–12): This questionnaire evaluates psychological distress using 12 four-point Likert scale questions [20]. Each item is rated on a four-point scale (less than usual, no more than usual, rather more than usual, or much more than usual). For the present study, bi-modal scoring method was chosen, transforming individual items into dichotomous (0–-0-1-1) and adding these without weighting into the GHQ score (range 0–12). This method was selected to eliminate any bias resulting from the tendency of the respondents to choose answers 1 and 4 or 2 and 3 [21]. The cut-off point of 3 implied those with a score of 3 or more may be experiencing psychological distress [22].

In addition, two indices were developed specifically for this study, the Spinal Stiffness Index and the Functional Limitation Index. The items and categories selected to construct both instruments are intended to evaluate the impact of the axSpA on the daily life of patients from their perspective. Said indexes were developed by the multi-stakeholder steering committee of the project during the survey development phase, which was responsible for ensuring its content validity.

#### Spinal stiffness index

This index, developed specifically for this study, assesses the degree of stiffness experienced by patients in the spinal column, distinguishing between the cervical, dorsal and lumbar areas. Possible responses range from least to most affected column (1: without stiffness, 2: mild stiffness, 3: moderate stiffness and 4: severe stiffness), total scores are obtained by adding together the responses in each of the areas of the spine without weighting resulting in a scale ranging from 3 to 12. This index showed an acceptable construct validity (Cronbach’s alpha = 0.79).

#### Functional limitation index

This index, developed specifically for this study, assesses the degree of functional limitation in 18 activities of daily life (dressing, grooming, bathing, tying shoelaces, moving about the home, walking up and down stairs, getting into/out of bed, using the toilet, shopping, preparing meals, eating, housework, walking, using public transportation, going to the doctor, driving, physical exercise and engaging in intimate relations). Each of these 18 activities was assigned as 0 for no limitation, 1 low limitation, 2 medium limitation and 3 high limitations, resulting in values between 0 and 54. A total score from 0 and 18 was considered low limitation, between 18 and 36 medium limitation and between 36 and 54 high limitation. Cronbach alpha of 0.97 demonstrated excellent construct validity.

### Sample selection and recruitment

European unselected patients with a self-reported diagnosis of axSpA (r-axSpa or nr-axSpA), aged ≥ 18 years, having visited a healthcare professional for axSpA in the 12 months prior to participation were included in the survey.

**Fig. 1:**
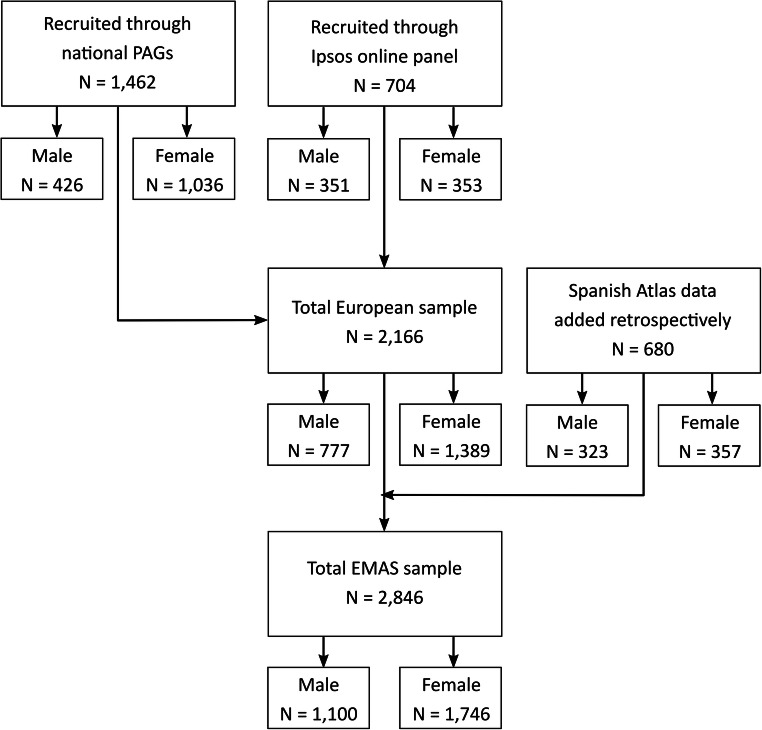
EMAS sample recruitment flowchart stratified by gender. This figure represents the sample selection flow chart for this study. A total of 1462 patients were recruited through national patient organizations, of which 426 were male and 1036 female. An additional 704 were recruited through Ipsos (known as Gfk at the moment of the survey) online panel, of which 351 were male and 353 females. Total European sample was composed of 2166 individuals (777 males and 1389 females). To that number, the database of the Atlas of Axial Spondyloarthritis in Spain was retrospectively added, composed of 680 individuals (323 males and 357 females). Total EMAS sample was composed of 2846 participants (1100 males and 1746 females). PAGs Patient Advocacy Groups, EMAS European Map of Axial Spondyloarthritis

Participants were recruited between July 2017 and March 2018 by the research agency Ipsos SA, formerly GfK, through their existing online patient panel. This firm ensures that patients are fully vetted through their contacted healthcare professionals around the world, who refer their patients to patient market research. The database from the Atlas of Axial Spondyloarthritis in Spain 2017 [17] was retrospectively added to the EMAS database. In Austria, France, Spain, Norway, Slovenia, Sweden, the Netherlands, Italy, Russia and Switzerland, axSpA-specific patient organizations also supported recruitment by distributing the survey to their associated members (Fig. 1).

All patients agreed to their participation through informed consent and were asked to provide explicit opt-in consent prior to participating in the EMAS survey. Participant data were anonymised.

### Statistical analysis

Sociodemographic variables analysed were age, marital status, and educational level; life style variables included smoking, alcohol consumption, physical activity and membership to patient organization; disease-related variables comprised different diagnostic journey milestones (age at onset of first symptoms, age at diagnosis, healthcare professionals—HCPs—seen before diagnosis), pharmacological and alternative treatments, as well as the presence of a family history of axSpA, HLA-B27 positivity, and extra-articular manifestations (uveitis, inflammatory bowel disease). Finally, patient-reported outcomes like disease activity (BASDAI), self-reported stiffness (Spinal Stiffness Index), limitation in daily life activities (Functional Limitation Index), and psychological distress (12-item General Health Questionnaire; GHQ-12) were also introduced. Selected variables were compared between male and female patients using the Mann-Whitney, Kruskal-Wallis and chi-square tests to assess the statistical significance of the observed differences between both groups. Results were reported as mean and standard deviation for continuous variables and as frequency and percentage for categorical variables. Statistical analysis was performed using SPSS version 25.0, and significance was set at 0.05. Methodology of the EMAS study is further described in its introductory article. [23]

## Results

### Sample description

**Fig. 2:**
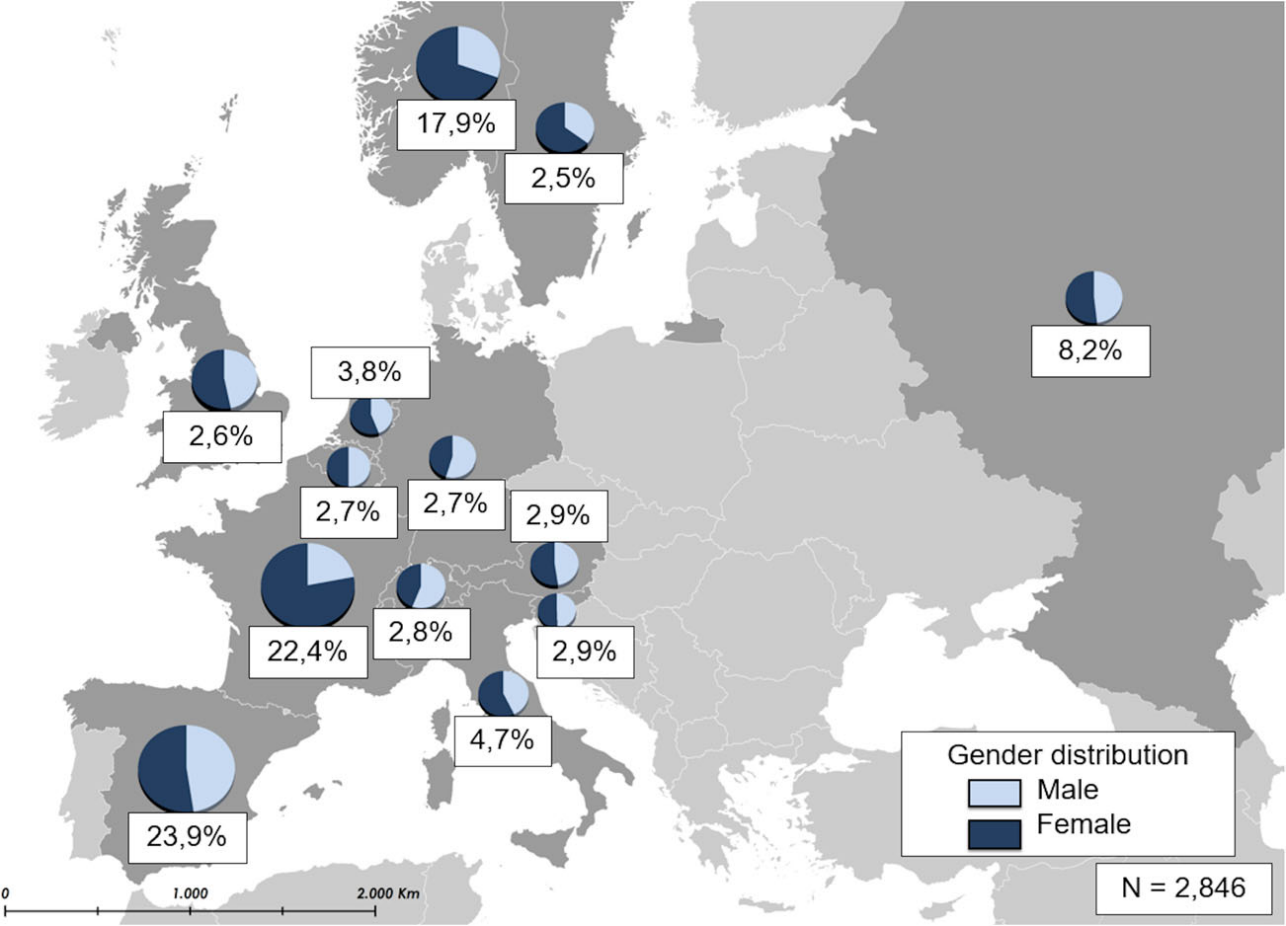
European sample distribution of patients for each EMAS country stratified by gender (*N* = 2846). The depicted European map represents the contribution of each EMAS participating country to the total sample size of 2846 participants. France provided one of the largest proportions (22.4%), followed by Spain (23.9%), Norway (17.9%), and the Russian Federation (8.2%). Italy provides 4.7%, the Netherlands 3.8%, Austria and Slovenia 2.9%, Switzerland 2.8%, Belgium and Germany 2.7%, the UK 2.6% and Sweden 2.5%. Gender distribution, as shown in the individual pie charts, is similar among countries with the exception of France, Norway, and Sweden (with a majority of female participants). The proportion of males was only slightly larger among the Swiss and German samples. Percentages represent each country’s contribution to the total sample. Pie charts represent the gender distribution of each country.

A total of 1100 (38.7%) males and 1746 (61.3%) females participated in the EMAS, with similar gender distribution across most countries (Fig. 2).

### Sociodemographic and lifestyle

Compared with men, women from the sample were significantly younger (more than 3 years on average). Women had more university-level studies and were less likely to be married. In addition, women more frequently reported to be homemakers or to be on temporary sick leave while male patients were more likely to be retired (Table 1). Men were more commonly members of a patient association. Regarding lifestyle habits, women were less prone to be regular alcohol users and smoke, but they also were less likely to engage in physical exercise than men were.

**Table 1:** Sociodemographic and lifestyle characteristics stratified by gender (*N* 2846, unless otherwise specified)

	Men (*n* 1100) (mean ± SD or %)	Women (*n* 1746) (mean ± SD or %)	*p* value
Sociodemographic characteristics			
Age (years)	46.1 ± 13.1	42.6 ± 11.5	**< 0.001**
Marital status (married)	787 (71.5)	1146 (65.6)	**< 0.001**
Educational level (university)	505 (45.9)	865 (49.5)	**< 0.001**
Employment status of active population*, *n* 1653			
- Employed	596 (89.2)	854 (86.7)	0.125
- Unemployed	72 (10.8)	131 (13.3)	0.125
Employment status of economically inactive*, *n* 1042			
- Temporary sick leave	85 (21.6)	219 (33.8)	**< 0.001**
- Permanent sick leave	112 (28.4)	180 (27.8)	0.821
- Retired	142 (36.0)	88 (13.6)	**< 0.001**
- Student	19 (4.8)	40 (6.2)	0.360
- Homemaker	11 (2.8)	103 (15.9)	**< 0.001**
Lifestyle habits			
Smoker (yes), *n* 2751	372 (35.0)	528 (31.3)	**0.040**
Alcohol (≥ 1 per week), *n* 2751	266 (25.0)	259 (15.3)	**< 0.001**
Physical exercise	938 (85.3)	1391 (79.7)	**< 0.001**
Patient organization membership	459 (41.7)	648 (37.1)	**0.014**

*Active population included the employed and unemployed of working age (15–64 years old). Participants who reported being on temporary sick leave, permanent sick leave, retired, having taken early retirement or being a student or homemaker were considered part of the inactive population

Bold values denote statistical significance at the *p* < 0.05 level

### Patient journey to axSpA diagnosis

Compared with males, females reported substantially longer diagnostic delay (6.1 ± 7.4 vs 8.2 ± 8.9; < 0.001), shorter disease duration (16.1 ± 11.7 vs 18.9 ± 13.3), and higher number of visits before being diagnosed to general practitioners (74.7 vs 82.1; < 0.001), osteopaths (13.3% vs 24.4%; < 0.001), and physiotherapists (34.5% vs 49.5%; *p* < 0.001). Females also reported a lower frequency of HLA-B27 carriership (80.2% vs 66.7%; < 0.001). Additionally, female patients had also been treated more frequently with pharmacological therapies (NSAIDS and biologics) and more with alternative therapies, as compared to males (Table 2).

**Table 2:** Patient journey and treatment stratified by gender (*N* 2846, unless otherwise specified)

	Men (*n* 1100)(mean ± SD or %)	Women (*n* 1746)(mean ± SD or %)	*p* value
Disease characteristics			
Age at onset of first symptoms (years), *n* 2721	27.0 ± 11.8	26.4 ± 10.7	0.342
Age at diagnosis (years), *n* 2722	32.6 ± 12.2	34.4 ± 10.9	**< 0.001**
Diagnostic delay (years), *n* 2652	6.1 ± 7.4	8.2 ± 8.9	**< 0.001**
Disease Duration (years), *n* 2716	18.9 ± 13.3	16.1 ± 11.7	**< 0.001**
HCP seen before diagnosis			
- General practitioner	822 (74.7)	1434 (82.1)	**< 0.001**
- Orthopaedic specialist	377 (34.3)	557 (31.9)	0.190
- Physiotherapist	380 (34.5)	865 (49.5)	**< 0.001**
- Osteopath, *n* 2166	103 (13.3)	339 (24.4)	**< 0.001**
- Other, *n* 2220	135 (14.0)	233 (18.5)	**0.005**
Family history of axSpA (yes), *n* 2244	291 (33.5)	584 (42.5)	**< 0.001**
HLA-B27 (positive), *n* 1799	497 (80.2)	786 (66.7)	**< 0.001**
Uveitis (yes), *n* 2096	199 (25.2)	270 (20.7)	**0.023**
Inflammatory bowel disease (yes), *n* 2096	113 (14.3)	181 (13.9)	0.688
Treatment			
Pharmacological treatment			
- NSAIDs (without biologics), *n* 2319	324 (34.7)	533 (38.5)	0.068
- Biologics (with or without NSAIDs), *n* 2316	359 (38.4)	594 (43.0)	**0.024**
Alternative treatment (acupuncture, homoeopathy, etc.) (yes), *n* 2170	268 (34.3)	541 (38.9)	**0.032**

Bold values denote statistical significance at the *p* < 0.05 level

Women reported significantly higher disease activity than men by nearly all different aspects assessed within the BASDAI scale (except morning stiffness duration), along with greater functional limitation, higher risk of psychological distress (GHQ-12), and higher prevalence of affective disorders (anxiety and depression) (Table 3). In addition, women reported a higher functional limitation in a series of daily life activities including going to the doctor, housework, shopping, using public transportation, going up or down the stairs, walking down the street, cooking and getting around the house (*p* < 0.001; see Supplementary Table 1).

**Table 3:** Patient-reported outcomes stratified by gender(*N* 2846, unless otherwise specified)

	Men (*n* 1100)(mean ± SD or %)	Women (*n* 1746)(mean ± SD or %)	*p*
Patient-reported outcomes (PROs)			
BASDAI (0–10) *n* 2584	5.1 ± 2.0	5.7 ± 1.9	**< 0.001**
- Fatigue, *n* 2636	5.7 ± 2.4	6.6 ± 2.2	**< 0.001**
- Neck, back or hip pain, *n* 2636	5.6 ± 2.4	6.2 ± 2.2	**< 0.001**
- Pain other than neck, back or hip, *n* 2636	4.3 ± 2.7	4.9 ± 2.6	**< 0.001**
- Discomfort to touch or pressure, *n* 2636	4.5 ± 2.7	5.6 ± 2.6	**< 0.001**
- Morning stiffness level, *n* 2636	5.3 ± 2.6	5.9 ± 2.6	**< 0.001**
- Morning stiffness duration, *n* 2584	4.5 ± 2.8	4.7 ± 2.8	0.070
Stiffness (3–12), *n* 2707	7.7 ± 2.6	7.8 ± 2.4	0.107
Functional limitation (0–54), *n* 2771	19.1 ± 16.7	21.2 ± 16.0	**< 0.001**
GHQ-12*, *n* 2640	4.4 ± 4.2	5.3 ± 4.1	**< 0.001**
GHQ-12* ≥ 3	564 (55.4)	1060 (65.4)	**< 0.001**
Diagnosis of anxiety	243 (30.6)	566 (43.3)	**< 0.001**
Diagnosis of depression	238 (30.1)	472 (36.1)	**< 0.001**

*GHQ-12:* 12-item General Health Questionnaire

*A value ≥ 3 implies a risk of psychological distress

Bold values denote statistical significance at the *p* < 0.05 level

## Discussion

The results of this large sample show substantial differences in how males and females with axSpA experience the disease. First, the journey to diagnosis of axSpA may be much longer and arduous for females, as reported by patients. EMAS female participants not only experienced 2-year longer diagnostic delay than males but also reported visiting general practitioners, osteopaths, and physiotherapists before being diagnosed to a greater extent than their male counterparts. The greatest gender difference was observed in physiotherapist visits prior to diagnosis, with half of females attending physiotherapists as compared with a third of males. As suggested in previous studies [4], gender differences in diagnosis of axSpA could be due to physicians underestimating women’s symptoms, resulting in inappropriate referrals to physicians other than a rheumatologist. Another potential reason for the longer delay in diagnosis could be lower frequency of HLA-B27 carriership among female participants. HLA-B27 positivity is a core feature within the ASAS classification criteria of axSpA in patients who do not show evidence of sacroiliitis on MRI scan [24]. Nevertheless, females frequently reported a positive family history of SpA which could help to identify axSpA, but is not one of the key features [24].

Failure to diagnose axSpA at an early stage means lack of an adequate treatment leading to continued pain, stiffness and fatigue, alongside a potential progressive loss of spinal mobility and function [25]. The fact that EMAS results show women experiencing a longer diagnostic delay could explain the poorer patient-reported outcomes of female participants.

As demonstrated in previous studies [2, 26], the results from EMAS also showed that females reported higher disease activity, functional limitation and mental health impact compared with males. Differences on disease activity were shown across almost all BASDAI items including fatigue, neck, back or hip pain, pain other than neck, back or hip, discomfort to touch or pressure and morning stiffness level. Furthermore, women reported a higher degree of psychological distress as measured by the GHQ-12 and more frequently reported a diagnosis of depression and anxiety. Psychological distress has been demonstrated to lead to poorer disease outcomes by enhancing symptom burden, decreasing adherence to treatment and increasing disability [27]. Additionally, in patients with axSpA, the risk of mood disorders is explained mainly by the degree of disease activity [28].

Women also reported significantly higher functional limitation when going to the doctor, executing housework or cleaning, shopping, cooking, walking or getting around the house, using public transportation, going up or down the stairs, and walking down the street, which is not surprising if we take into account that women were more likely homemakers compared to males.

AxSpA is a disease that can affect several areas of an individual’s life. A status of high disease activity, which for the patient means continuous pain, stiffness and fatigue, limits the performance of both professional and leisure activities [29]. At the same time, it prevents the patient from concentrating and enjoying those tasks that could be achievable, eliciting frustration, hindering feelings of accomplishment, and producing psychological distress [30, 31]. This way, the overall quality of life of people with axSpA is compromised [32]. For this reason, as EMAS results show how the female gender is associated with poorer disease outcomes - in the form of greater disease activity, functional limitation, and psychological distress among others-, it could be inferred that women with axSpA see their quality of life even more severely compromised than their male counterparts.

EMAS is the survey that has included the largest number of countries (13 European nations), gathering 2846 respondents. The inclusion of information from a sample of axSpA patients from countries as varied as Spain, Norway or Russia has served to capture differences across the European continent, generally ignored in previous published studies. The EMAS focus was on understanding the patient perspective through a holistic approach and utilizing a questionnaire designed for patients, with patients. However, this study is not without limitations. First, all data collected for the EMAS study was patient reported, and therefore, information relating to the time period preceding the year prior to the survey may be subject to recall bias. In addition, participants were asked to self-report a clinician-given diagnosis of an axSpA-related disease in order to participate in the survey. However, there were no attempts to further confirm participants’ responses with clinician-reported assessments. Second, it is important to bear in mind that the survey includes scales or indices for assessing certain factors that are yet to be validated. The reason for this action is grounded in the EMAS core values of patient participation in the study. These indices originated during the preliminary phase of the survey development, when patients expressed their concern that other validated scales or indices were unable to capture the whole scope of their everyday experience. In any case, Cronbach’s alpha coefficient for the used indexes was satisfactory, which testifies to the reliability of these instruments. Finally, the possibility of selection bias cannot be ruled out for those countries that included a smaller sample size.

## Conclusions

Important gender differences were observed among axSpA patients with respect to the journey to diagnosis, disease-related outcomes and psychological burden using a survey sample of 2846 patients with axSpA across 13 European countries. The path to diagnosis of axSpA seems to be longer and arduous in females, who also experience higher disease activity and poorer psychological health.

Overall, the difficulties faced by female EMAS participants highlight the need for further education among physicians to ensure that historical biases or differences in the presentation of the disease do not negatively affect the diagnosis and management of female axSpA patients, and thereby ensure equal access to care and optimal health outcomes for both men and women.

## Supplementary information

ESM 1 (DOCX 17.7 kb).

## Data Availability

All data collected by the EMAS study will be available in the white report “European Map of Axial Spondyloarthritis” (publication pending).

## References

[1] Sieper J, Poddubnyy D (2017). Axial spondyloarthritis. Lancet.

[2] Rusman T, van Vollenhoven RF, van der Horst-Bruinsma IE (2018). Gender differences in axial spondyloarthritis: women are not so lucky. Curr Rheumatol Rep.

[3] Jovaní V, Blasco-Blasco M, Ruiz-Cantero MT, Pascual E (2017). Understanding how the diagnostic delay of spondyloarthritis differs between women and men: a systematic review and metaanalysis. J Rheumatol.

[4] Jovani V, Blasco-Blasco M, Pascual E, Ruiz-Cantero MT (2018). Challenges to conquer from the gender perspective in medicine: the case of spondyloarthritis. PLoS One.

[5] Polley HF, Slocumb CH (1947). Rheumatoid spondylitis: a study of 1,035 cases. Ann Intern Med.

[6] West HF (1949). Aetiology of ankylosing spondylitis. Ann Rheum Dis.

[7] Kennedy LG, Will R, Calin A (1993). Sex ratio in the spondyloarthropathies and its relationship to phenotypic expression, mode of inheritance and age at onset. J Rheumatol.

[8] Masi AT, Wilkins WR (1996). Does male:female sex ratio in ankylosing spondylitis change with age?. J Rheumatol.

[9] Braun J, Sieper J (2007). Ankylosing spondylitis. Lancet..

[10] López-Medina C, Moltó A (2018). Update on the epidemiology, risk factors and disease outcomes of axial spondyloarthritis. Best Pract Res Clin Rheumatol.

[11] Siar N, Akasbi N, Zoukal S, Ghazzali S, el Kohen K, Harzy T (2019) Axial spondyloarthritis in women: differences in disease onset, clinical presentation, and disease activity and functional indices. Curr Rheumatol Rev 15. 10.2174/1573397115666190626113230

[12] Huang WN, Tso TK, Kuo YC (2012). Distinct impacts of syndesmophyte formation on male and female patients with ankylosing spondylitis. Int J Rheum Dis.

[13] Ortolan A, van Lunteren M, Ramiro S, Ramonda R, Landewé RBM, Dagfinrud H, Jacobsson LTH, van der Heijde D, van Gaalen FA (2018). Are gender-specific approaches needed in diagnosing early axial spondyloarthritis? Data from the spondyloarthritis caught early cohort. Arthritis Res Ther.

[14] Van Der Horst-Bruinsma IE, Zack DJ, Szumski A (2013). Female patients with ankylosing spondylitis: analysis of the impact of gender across treatment studies. Ann Rheum Dis.

[15] Swinnen TW, Westhovens R, Dankaerts W, de Vlam K (2018). Widespread pain in axial spondyloarthritis: clinical importance and gender differences. Arthritis Res Ther.

[16] Wroński J, Fiedor P, Głuszko P (2019). Adverse events in patients with ankylosing spondylitis treated with TNF inhibitors: a cross-sectional study. Int J Clin Pharm.

[17] Garrido-Cumbrera M, Navarro-Compán V, Zarco P, Collantes-Estévez E, Gálvez-Ruiz D, Braçe O, Chacón García J, Blanch Mur C, Costa Ferrer A, Hidalgo Vega A, Plazuelo Ramos P, Gratacós Masmitja J (2019). Atlas of axial spondyloarthritis in Spain 2017: study design and population. Reumatol Clin.

[18] Garrido-Cumbrera M, Gálvez-Ruiz D, Chacón García J et al (2017) Atlas of axial spondyloarthritis in Spain 2017*.* Profile of the disease*.* Max Weber Institute, Madrid

[19] Garrett S, Jenkinson T, Kennedy LG, Whitelock H, Gaisford P, Calin A (1994) A new approach to defining disease status in ankylosing spondylitis: the Bath Ankylosing Spondylitis Disease Activity Index. J Rheumatol 21:2286–2291 7699630

[20] Goldberg D, Williams P (1988) A user’s guide to the General Health Questionnaire. Windsor: NFER-Nelson

[21] Goldberg DP, Gater R, Sartorius N (1997). The validity of two versions of the GHQ in the WHO study of mental illness in general health care. Psychol Med.

[22] Cano A, Sprafkin RP, Scaturo DJ, Lantinga LJ, Fiese BH, Brand F (2001). Mental health screening in primary care: a comparison of 3 brief measures of psychological distress. Prim Care Companion J Clin Psychiatry.

[23] Garrido-Cumbrera M, Poddubnyy D, Gossec L (2019). The European Map of Axial Spondyloarthritis: capturing the patient perspective—an analysis of 2846 patients across 13 countries. Curr Rheumatol Rep.

[24] Rudwaleit M (2010). New approaches to diagnosis and classification of axial and peripheral spondyloarthritis. Curr Opin Rheumatol.

[25] Sieper J, Rudwaleit M (2005) Early referral recommendations for ankylosing spondylitis (including pre-radiographic and radiographic forms) in primary care:659–663. 10.1136/ard.2004.028753 10.1136/ard.2004.028753 PMC1755504 15528281

[26] Lee W, Reveille JD, Davis JC, Learch TJ, Ward MM, Weisman MH (2007). Are there gender differences in severity of ankylosing spondylitis? Results from the PSOAS cohort. Ann Rheum Dis.

[27] Kotsis K, Voulgari PV, Drosos AA, Carvalho AF, Hyphantis T (2014). Health-related quality of life in patients with ankylosing spondylitis: a comprehensive review. Expert Rev Pharmacoecon Outcomes Res.

[28] Garrido-Cumbrera M, Delgado-Domínguez CJ, Gálvez-Ruiz D, Mur CB, Navarro-Compán V (2019) The effect of axial spondyloarthritis on mental health: results from the Atlas. J Rheumatol 46:1284–1289. 10.3899/jrheum.180868 10.3899/jrheum.180868 30770507

[29] Packham J (2018). Optimizing outcomes for ankylosing spondylitis and axial spondyloarthritis patients: a holistic approach to care. Rheumatology (Oxford).

[30] Hamilton-West KE, Quine L (2009). Living with ankylosing spondylitis: the patient’s perspective. J Health Psychol.

[31] Bagcivan G, Cinar FI, Cinar M, Oflaz F, Uzun S, Pay S (2015). Living with pain in ankylosing spondylitis: a qualitative study. Contemp Nurse.

[32] Strand V, Singh JA (2017). Patient burden of axial spondyloarthritis. J Clin Rheumatol.

