# Supplementary material for: Gender differences in patient journey to diagnosis and disease outcomes: results from the European Map of Axial Spondyloarthritis (EMAS)
Source: Clin Rheumatol. 2021 Jan 19;40(7):2753–61. doi: 10.1007/s10067-020-05558-7 (PMC8189938; doi:10.1007/s10067-020-05558-7)
Supplement: Supplementary file 1 — (DOCX 17.7 kb). [file 10067_2020_5558_MOESM1_ESM.docx]

**Supplementary table 1.** Gender differences by functional limitation in daily activities (low, medium and high)

| **Functional limitation in daily activities** | **Gender** | | | | | | | | | | | | **P-value** |
| --- | --- | --- | --- | --- | --- | --- | --- | --- | --- | --- | --- | --- | --- |
|  | **Male** | | | | | | **Female** | | | | | |  |
|  | **Low** | | **Medium** | | **High** | | **Low** | | **Medium** | | **High** | |  |
|  | **n** | **%** | **n** | **%** | **n** | **%** | **n** | **%** | **n** | **%** | **n** | **%** |  |
| Going to the doctor | 103 | 32.0% | 128 | 39.8% | 91 | 28.3% | 63 | 18.4% | 111 | 32.5% | 168 | 49.1% | **<0.001*** |
| Housework / cleaning | 160 | 24.4% | 250 | 38.1% | 247 | 37.6% | 147 | 11.2% | 482 | 36.7% | 683 | 52.1% | **<0.001*** |
| Shopping | 176 | 29.8% | 209 | 35.4% | 206 | 34.9% | 196 | 16.8% | 415 | 35.6% | 554 | 47.6% | **<0.001*** |
| Using public transportation | 217 | 39.0% | 193 | 34.6% | 147 | 26.4% | 279 | 31.4% | 264 | 29.7% | 346 | 38.9% | **<0.001*** |
| Going up or down the stairs | 165 | 24.5% | 269 | 39.9% | 240 | 35.6% | 228 | 18.7% | 430 | 35.2% | 562 | 46.1% | **<0.001*** |
| Walking down the street | 169 | 27.5% | 243 | 39.6% | 202 | 32.9% | 234 | 21.9% | 381 | 35.6% | 455 | 42.5% | **<0.001*** |
| Cooking | 225 | 41.6% | 199 | 36.8% | 117 | 21.6% | 302 | 31.4% | 370 | 38.5% | 289 | 30.1% | **<0.001*** |
| Walking / getting around the house | 201 | 33.1% | 242 | 39.8% | 165 | 27.1% | 272 | 25.3% | 436 | 40.5% | 368 | 34.2% | **0.001*** |
| Driving | 200 | 32.5% | 243 | 39.4% | 173 | 28.1% | 313 | 30.1% | 379 | 36.4% | 348 | 33.5% | 0.075 |
| Engaging in intimate relations | 165 | 26.9% | 228 | 37.2% | 220 | 35.9% | 266 | 25.2% | 370 | 35.0% | 420 | 39.8% | 0.29 |
| Lying down / getting up from bed | 177 | 25.6% | 267 | 38.6% | 247 | 35.7% | 242 | 20.0% | 496 | 41.1% | 470 | 38.9% | 0.018 |
| Eating | 324 | 63.5% | 148 | 29.0% | 38 | 7.5% | 497 | 61.9% | 220 | 27.4% | 86 | 10.7% | 0.14 |
| Doing physical exercise | 116 | 15.2% | 271 | 35.6% | 375 | 49.2% | 184 | 14.1% | 445 | 34.2% | 674 | 51.7% | 0.528 |
| Going to the toilet | 229 | 41.6% | 208 | 37.8% | 113 | 20.5% | 382 | 42.7% | 324 | 36.2% | 189 | 21.1% | 0.826 |
| Dressing / undressing | 250 | 33.6% | 294 | 39.5% | 201 | 27.0% | 388 | 33.3% | 475 | 40.8% | 301 | 25.9% | 0.807 |
| Taking a bath / shower | 230 | 38.7% | 208 | 35.0% | 156 | 26.3% | 365 | 37.7% | 367 | 37.9% | 236 | 24.4% | 0.482 |
| Washing / personal grooming | 233 | 39.6% | 204 | 34.7% | 151 | 25.7% | 379 | 40.8% | 342 | 36.9% | 207 | 22.3% | 0.311 |
| Tying shoe laces | 172 | 23.5% | 270 | 36.9% | 290 | 39.6% | 288 | 25.7% | 433 | 38.7% | 398 | 35.6% | 0.199 |

* P < 0.001

Bold values denote statistical significance at the *p* < 0.05 level
